# Low host specificity and abundance of frugivorous lepidoptera in the lowland rain forests of Papua New Guinea

**DOI:** 10.1371/journal.pone.0171843

**Published:** 2017-02-23

**Authors:** Katerina Sam, Richard Ctvrtecka, Scott E. Miller, Margaret E. Rosati, Kenneth Molem, Kipiro Damas, Bradley Gewa, Vojtech Novotny

**Affiliations:** 1 University of South Bohemia, Faculty of Science, Branisovska, Ceske Budejovice, Czech Republic; 2 Biology Centre CAS, Institute of Entomology, Branisovska, Ceske Budejovice, Czech Republic; 3 National Museum of Natural History, Smithsonian Institution, Washington, United States of America; 4 New Guinea Binatang Research Center, Madang, Papua New Guinea; 5 The University of Papua New Guinea, Waigani, University, National Capital District, Papua New Guinea; 6 Papua New Guinea Forest Research Institute, Lae, Morobe Province, Papua New Guinea; University of Arkansas, UNITED STATES

## Abstract

We studied a community of frugivorous Lepidoptera in the lowland rainforest of Papua New Guinea. Rearing revealed 122 species represented by 1,720 individuals from 326 woody plant species. Only fruits from 52% (171) of the plant species sampled were attacked. On average, Lepidoptera were reared from 1 in 89 fruits and a kilogram of fruit was attacked by 1.01 individuals. Host specificity of Lepidoptera was notably low: 69% (33) of species attacked plants from >1 family, 8% (4) fed on single family, 6% (3) on single genus and 17% (8) were monophagous. The average kilogram of fruits was infested by 0.81 individual from generalist species (defined here as feeding on >1 plant genus) and 0.07 individual from specialist species (feeding on a single host or congeneric hosts). Lepidoptera preferred smaller fruits with both smaller mesocarp and seeds. Large-seeded fruits with thin mesocarp tended to host specialist species whereas those with thick, fleshy mesocarp were often infested with both specialist and generalist species. The very low incidence of seed damage suggests that pre-dispersal seed predation by Lepidoptera does not play a major role in regulating plant populations via density-dependent mortality processes outlined by the Janzen-Connell hypothesis.

## Introduction

Lepidoptera represent an important component of the frugivorous insect guild, but have rarely been studied at a community level [1,2]. Many studies [3–6] are restricted to single lepidopteran families, and/or to a single plant family [7–9] or even a single plant species [10–13].

Although we know that many frugivorous Lepidoptera have an effect on fruits of economically important plants [14], their role in tropical forests is almost unknown because of the lack of life history information on rainforest Lepidoptera species, sampled often only as adults. Fruits represent two very distinct, high-quality food resources: seeds and mesocarp [15,16] and can therefore be attacked by seed predators [8,9,17] as well as frugivores attacking mesocarp [11] or the whole immature fruit content [18]. In each of these cases caterpillars negatively affect fruit development and cause fruit abortion or early fruit fall, but the importance of this impact on fruit production and survival remains unknown. Moreover, some of lepidopterans can be scavengers of aborted fruits on the ground [19].

The most important herbivores affecting seed survival are seed predators as they decrease plant reproductive potential [20,21]. It has been shown before that insect-damaged fruits were rejected by birds (i.e. dispersers) and increased abundance of damaged fruits led also to increased rejection of undamaged fruits carried by the infested shrubs [22]. Lepidoptera, as well as other frugivorous insects, may cause seed mortality as well as dispersal failure and contribute thus to maintaining high species diversity in tropical forest, particularly if seed mortality were positively dependent on plant density [23–25]. Such density dependence is more likely in specialized herbivores than in generalists.

Our goals are to examine the abundance, species richness and host specificity of frugivorous Lepidoptera (while other frugivorous insects were studied separately [26,27]) on a phylogenetically diverse sample of plants in the lowland rain forest of Papua New Guinea. We test if they are (i) host specific, (ii) causing seed mortality that could potentially affect population dynamics and size of plant species, (iii) potentially important density-dependent mortality factors in maintaining plant diversity and (iv) responding to fruit and seed morphology.

## Materials and methods

### Study areas

We conducted the study in two areas approximately 100 km apart: (1) near the villages of Baitabag, Mis and Ohu within a 20 × 10-km area comprising a successional mosaic of disturbed and mature lowland rainforest (5°08'-14'S, 145°7'-41'E, 50–200 m asl, Madang Province, Papua New Guinea), and (2) in relatively less disturbed forest near Wanang village (5°14'S, 145°11'E, 100 m asl), in the proximity of CTFS plot [26]. The lands at the four study sites were privately owned, legally recognized by the PNG Government as lands under indigenous tribal ownership. The access for sampling was approved by the relevant communities led by Hais Wasel (Ohu Village), John Auga (Mis Village), Kiatik Batet (Baitabag) and Filip Damen (Wanang). Field studies did not involve any endangered or protected species. Vegetation at the four study sites is very similar in species composition [26], classified as mixed evergreen rain forest on Latosol [28–30] with a humid climate (mean annual rainfall 3,600 mm), a mild dry season from July to September, and mean annual temperature of 26°C [31]. The study sites have been described in detail by Ctvrtecka *et al*. [26].

### Study design

We conducted the all-yearlong study between March 2008 and April 2009. Fruits were sampled by searching a 200–400-ha matrix of mature and early-successional forest at each site and by collecting all plant species encountered in the fruiting condition. The methodology was described by Ctvrtecka *et al*. [26,27]. Sampling effort amounted to the total of 1,284 person-days of field work during which mature or nearly mature fruits were collected. Fresh fruits from branches (68% of fruit pieces, 66% of fruit biomass and 68% of samples), the forest floor (31% pieces, 32% biomass and 30% of samples) or from unknown origin (1–2%, either branch or forest floor) were collected whereas decomposing fruits on the ground were avoided. Origin of the sample had no effect on the infestation rate (F_2,226_ = 0.85, P = 0.428). Individual sample (collection of fruits from an individual tree or liana on a particular day) comprised from 1 to 1500 individual fruits and weighed between 22 and 8311 g [26,27]. We employed a functional definition of the individual fruit that encompassed aggregate fruits arising from the fusion of adjacent carpels (e.g. *Artocarpus* and *Ficus*). For a subset of plant species, basal area in a 50-ha CTFS plot in Wanang, where all individual trees with dbh >1 cm were measured and identified [32,33] was used as a proxy for local abundance [26,27]. Plot data allowed us to calculate basal area for 218 species out a total of sampled 531 plant species.

One or several ripe fruits from each sample were cut along two perpendicular axes and photographed [26,27]. Cross-sectional area of the fruit and the seed were estimated for 268 species. Measurements on the photographs were made in Adobe Photoshop and the volume of each was calculated as a volume of ellipsoid (4/3 × π ×A/2 x B/2 x C/2, where A, B and C is the length, width and height of the fruit or seed respectively) [26,27]. The volume of the fruit, the combined volume of seeds per fruit (in the case of many-seeded fruits), and fleshiness (% of fruit volume represented by mesocarp) were used as plant traits in an analysis of fruit suitability for caterpillar development.

Fruit-feeding caterpillars were reared in ventilated plastic boxes. Emerging adults were drawn to light through a drilled hole on the side of boxes and collected in vials that were collected every 24 h [26,27]. Often, we were not able to ascertain whether our reared lepidopteran species fed on mesocarp or seeds but their impact on fruit fitness may be important in both cases. Each fruit sample was reared for 10 weeks, which was deemed sufficient based on our previous experience with rearing other insect larvae from fruits and caterpillars from other plant parts [26,27]. All specimens were assigned to morphospecies using a reference collection in The New Guinea Binatang Research Center.

In addition to morphotyping, we attempted to obtain DNA sequences for five specimens from each species where available, then sampled additional specimens as needed to clarify variation [34–36]. DNA sequencing (COI barcode) followed standard methods at the Biodiversity Institute of Ontario, University of Guelph [34,35], using legs and the LepF1 and LepR1 primers. 540 vouchers were sampled for DNA, resulting in 490 successful sequences, a 91% success rate. These sequences group into 142 barcode clusters, using the RESL algorithm as implemented in BOLD [36]. We selectively dissected genitalia of over 50 specimens to compare genitalia to type specimens.

Identifications relied on the literature, voucher specimens in the Smithsonian National Museum of Natural History and Natural History Museum, London, and the BOLD identification tool, comparing our data to over 18,000 sequences generated for New Guinea Lepidoptera [37], and a larger library for Australian Lepidoptera, including more than 40,000 specimens from the Australian National Insect Collection [38]. Many species remain unidentified, and we welcome comments on all identifications. Where taxonomic names are not readily available from existing literature, BINs (DNA cluster-based morphospecies) can be used as species hypotheses that can be confirmed by future taxonomic studies [36,39]. Many of these records represent undescribed species, and we have purposefully refrained from assigning new names until the relevant taxa can be studied in sufficient detail. Under the Fort Lauderdale principles for genetic data [40] we ask others to refrain from assigning new species names to these records outside of the context of proper systematic study.

Data released on Genbank (accession numbers GU695412-5, GU695431-2, GU695434-66, GU695468-9, GU695504-46, GU695548-58, GU695561, GU695575-80, GU695623-36, GU695639-701, GU695716-7, GU695720-1, GU695745, HM376367-75, HM376381-4, HM422448-56, HM902704-15, HQ947496-7, HQ956600-1, HQ956613-4) include the standard fields for the BARCODE data standard [41] while more data, including images and host plants, are available on BOLD (www.boldsystems.org; [42]), accessible from the project FRUT using a DOI (dx.doi.org/10.5883/DS-LEPFRNG1).

Insect vouchers were deposited at the Smithsonian Institution and at the Papua New Guinea Agriculture Research Institute in Port Moresby. Fruit and plant vouchers were deposited at the Papua New Guinea Forest Research Institute in Lae and at the University of Minnesota in St. Paul. Digital photographs and voucher information associated with fruit specimens were submitted to the New Guinea Atrium digital herbarium (http://ng.atrium-biodiversity.org/atrium).

### Data analysis

Only fruits of plant species with a total sample weight of ≥1 kg and >50 individual fruits (N = 326 species) were included in the analyses (see [26] for more details). These thresholds represent a compromise between maximizing the number of plant species analysed and the thorough sampling of Lepidoptera assemblages from every host plant species [26]. Our sampling protocol did not monitor whether the collectors sampled fruits from unique tree individuals every time, or visited trees repeatedly on different sampling days. Taking into account that samples collected from the same plant species from the same study site within 30 days might be not be independent, we estimated that the 4,268 fruit samples were collected from at least 2,228 tree individuals.

Species accumulation analyses were based on complete Lepidoptera records including rare species and singleton records. However, host specificity was analysed for only those species represented by at least 10 individuals in our samples. Their host associations were considered only if supported by at least two observations of feeding. Host specificity was categorized as monophagous (M) for species feeding on a single plant species, congeneric (CG), confamilial (CF), and allofamilial (AF) for species feeding on either >1 congeneric species, >1 confamilial genus, or >1 family, respectively. Monophagous and congeneric host ranges are hereafter referred to as specialists and the remaining two categories as generalists. We did not assess seed mortality directly but rather calculated the density of caterpillars per fruit and per unit mass of fruit as a proxy for mortality.

The overlap between species reared from fruit and those feeding on plant foliage was estimated using our existing data on caterpillars feeding on the foliage sampled at our study areas ([43,44], http://datadryad.org/resource/doi:10.5061/dryad.rg155q32). Accumulation curves for herbivore species with increasing numbers of plant species and samples were implemented in EstimateS. The species richness of Lepidoptera was extrapolated for the local plant diversity using a power function fitted to the empirical data for N = 100–169 plant species [44].

## Results

In total, we collected 4,268 samples weighing 3,556.8 kg from 531 woody plant species representing 84 plant families. The frugivorous communities reared from this full dataset comprised 2,050 Lepidoptera adults from 151 species (DNA of 142 spp. was successfully analysed) from the following families: Tortricidae (38 spp.), Tineidae (16 spp.), Pyralidae (14 spp.), Crambidae (13 spp.), Cosmopterigidae (8 spp.), Lecithoceridae (8 spp.), Gracillariidae (5 spp.), Oecophoridae (7 spp.), Gracillariidae (5 spp.), Nolidae (5 spp.), Gelechiidae (4 spp.), Lycaenidae (4 spp.), Erebidae (3 spp), Noctuidae (3 sp.), Blastobasidae (2 spp.), Brachodidae (1 sp.), Carposinidae (1 sp.), Heliodinidae (1 sp.), Immidae (1 sp.), Pterophoridae (1 sp.), Schreckensteiniidae (1 sp.), Thyrididae (1), and Xylorictidae (1 sp.). Further 11 species of Lepidoptera and 2 species of Microlepidoptera were not been identified to the family level (S1 Table).

The dataset used for community analysis included 2,758.8 kg fruits from 326 plant species and 58 families sampled as at least 50 fruits weighing at least 1 kg in aggregate per plant species. The total sample size per plant species varied from 1–65 kg and 50–7,166 fruits. The total weight and number of fruits collected per tree species was significantly correlated with its basal area, a proxy for local abundance (sample weight = 6,670 + 0.0565 × basal area, R^2^ = 0.22, P < 0.001; number of fruits = 687 + 0.0072 × basal area, R^2^ = 0.37, P < 0.001).

**Fig 1:**
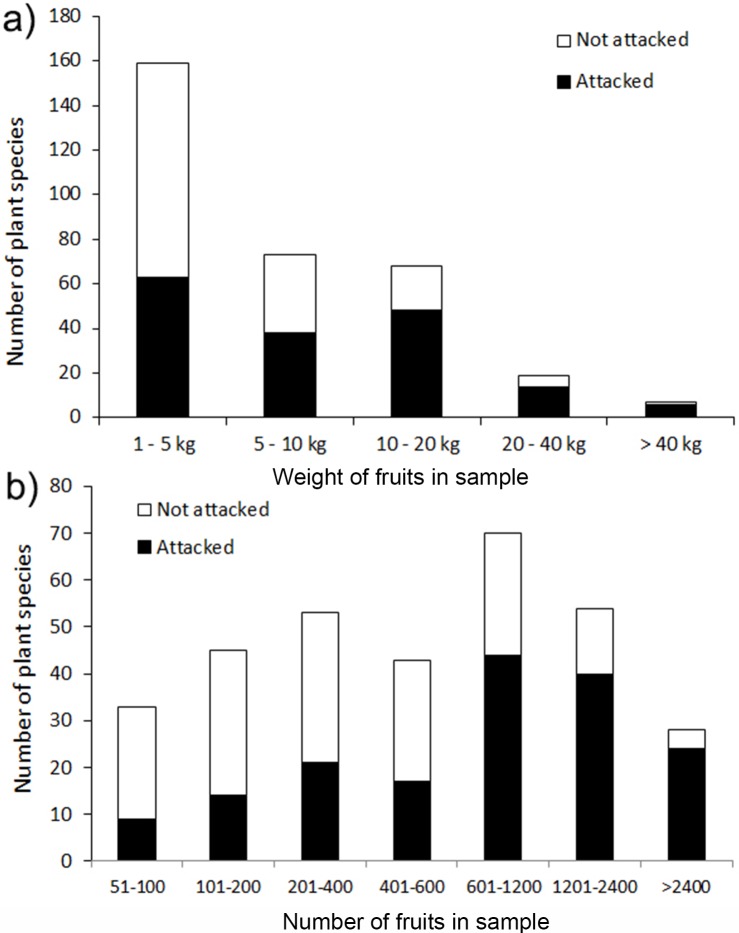
The number of plant species attacked (black bar) and not attacked (white bar) by frugivorous Lepidoptera in samples categorized by (a) fruit weight and (b) the number of fruits.

From this subset of 326 plant species we reared caterpillars from 171 plant species, i.e. 52.5% of species. The reared Lepidoptera included 1,720 individuals representing 122 species. The estimate of the proportion of plant species infested by lepidopterans increased with the total weight of the fruit sample (χ^2^_4_ = 47.4; P < 0.001; Fig 1a) and the number of fruits collected per species (Fig 1b).

Host specificity was quantified for the 48 Lepidoptera species represented by ≥10 individuals (S1 Table) reared from 169 plant species and 48 plant families. Thirty-three Lepidoptera species attacked plants from >1 family (allofamilial host range), four species attacked plants from >1 genus within a single family (confamilial host range), three species attacked >1 congeneric plant species and eight species were monophagous. Generalists were more abundant (mean ± SE = 0.81 ± 0.18 individuals kg^-1^ fruits) than specialists (mean ± SE = 0.07 ± 0.02 individuals kg^-1^ fruits), and than the species for which we were not able to determine their specialization (mean ± SE = 0.09 ± 0.02 individuals kg^-1^ fruits).

Overall, 14 from the total of 122 species reared from fruit had been previously found feeding on leaves (Table 1). These include three species where we could estimate their host specificity on fruits; they all were allofamilial.

**Table 1:** Overlap of Lepidoptera species in frugivorous (this study) and leaf-chewer (different study, [68]) guilds.

Family/species of Lepidoptera	Nf	Pf	S	Nl	Pl	Ns	Plant species
Cosmopterigidae/*Labdia* sp2	14	7	AF	1	1	1	*Ficus dammaropsis*
Crambidae/*Nacoleia octasema*	3	1		267	5	1	*Heliconia papuana*
Crambidae/*Pagyda salvalis*	4	2		142	3	1	*Vitex cofassus*
Crambidae/*Prophantis androstigmata*	4	3		1	1	1	*Randia decora*
Crambidae/*Prophantis* sp1	13	1		16	?	?	
Gracillariidae/*Conopomorpha cramerella*	16	3	AF	1	1	1	*Caesaria erythrocarpa*
Immidae/*Moca congrualis*	1	1		3	1	0	
Lycaenidae/*Deudorix epirus*	13	5	AF	7	?*	?	*Maniltoa*
Nolidae/*Etanna vittalis*	2	1		92	7	0	
Tortricidae/*Isodemis nr stenotera*	1	1		30	9	0	
Tortricidae/*Adoxophyes spn nr orana*	1	1		794**	88	0	
Tortricidae/*Adoxophyes* sp1	1	1		794**	88	0	
Tortricidae/*Adoxophyes tripselia*	1	1		164	29	0	
Tortricidae/Heleanna nr physalodes	19	1		92	7	1	*Pimelodendron amboinicum*

Nf = number of individuals reared from fruits, Pf = number of host plant species used by frugivorous individuals, S = specificity (AF = allofamilial), Nl = number of individuals reared from leaves, Pl = number of host plant species used by folivorous individuals, Ns = number of plant species from which both resources (fruits, leaves) were used, Plant species = plant name of the species from which both resources (fruits, leaves) were used, *incomplete plant identification (genus), **one unidentified lepidopteran morphospecies.

**Fig 2:**
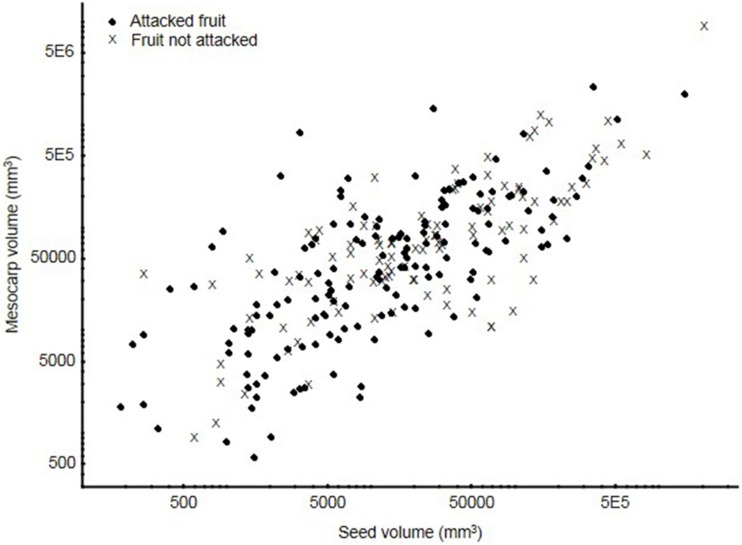
Relationship between seed and mesocarp volume for 268 plant species attacked and not attacked by Lepidoptera.

**Fig 3:**
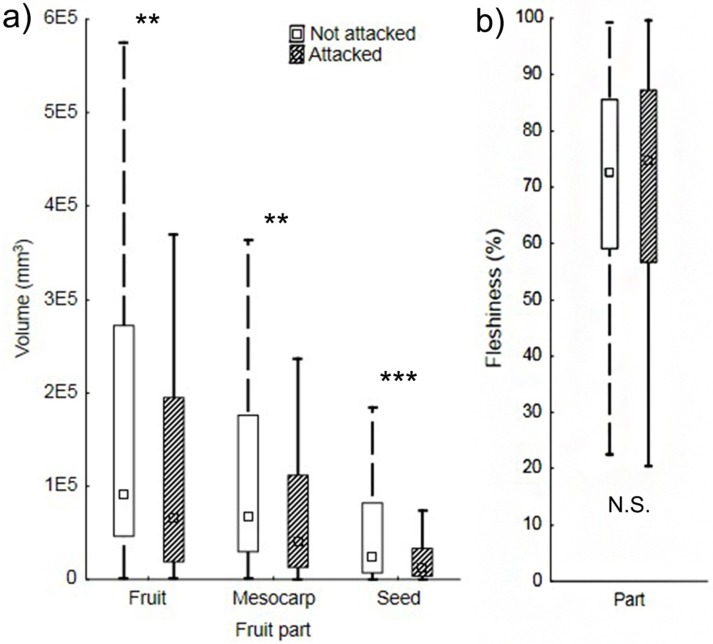
Mean volume for whole fruit, mesocarp and seeds (a) and fleshiness (b) in plant species attacked and not attacked by Lepidoptera. The differences between attacked and non-attacked species are significant (whole fruit: U _106,151_ = 5624, Z = 3.064, P = 0.002; mesocarp: U _106,151_ = 5828, Z = 2.69, P = 0.007; seeds: U _106,151_ = 5346, Z = 3.574, P < 0.0010), (b) Fleshiness (i.e. proportion of mesocarp in whole fruit) did not have significant effect on infestation (U _106,151_ = 6839, Z = 0.83, P = 0.401).

Mesocarp and seed volumes were significantly correlated across plant species [mesocarp = -27763.38 + 3.314*seed (mm^3^); r^2^ = 0.74], and plant species across the whole range of both mesocarp and seed sizes were infested (Fig 2). However, significantly smaller fruits, as well fruits with smaller volumes of seed and mesocarp were infested significantly more often than large fruits (fruit volume: U _106,151_ = 5624, Z = 3.064, P = 0.002; seeds volume: U _106,151_ = 5346, Z = 3.574, P < 0.001; mesocarp volume: U _106,151_ = 5828, Z = 2.69, P = 0.007). Fleshiness (i.e. proportion of mesocarp in whole fruit) differed significantly in infested vs. uninfested fruits (U _106,151_ = 6839, Z = 0.83, P = 0.401) (Fig 3).

**Fig 4:**
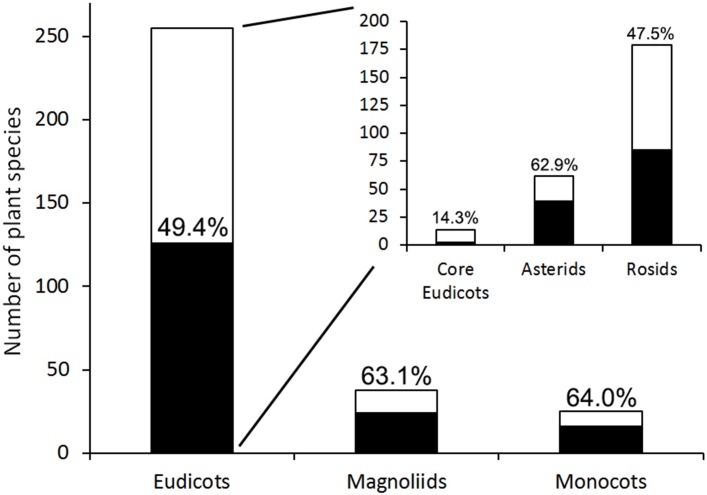
Numbers of Lepidoptera-infested plant species (black bars) relative to the total number of plant species sampled (white bars) for major flowering plant clades. The percentage of infested plant species is shown above the bars. The differences in attack rate between Magnoliids, eudicots and monocots were not significant. Within eudicots, Lepidoptera attacked more frequently asterids (63% of 62 species) than rosids (47% of 179 species, χ^2^_1_ = 3.8, P = 0.05), and core eudicots (14% of 14 species, χ^2^_1_ = 9, P = 0.002). Classification according to the Angiosperm Phylogeny Group (APG III 2009).

None of the Lepidoptera superfamilies or clades (Apodytrisia, Dytrisia, Macrolepidoptera, Obtectomera) showed any preferences towards specific fruit characteristics, including the whole fruit volume, flesh volume, and seed volume (superfamilies: H _6,267_ = 10.9–8.77, p = 0.09–0.51; clades: H _3,229_ = 6.07–3.91, p = 0.11–0.27). Magnoliids, eudicots and monocots were attacked with similar frequency. Within eudicots, Lepidoptera more frequently attacked asterids than rosids and core eudicots (Fig 4).

**Fig 5:**
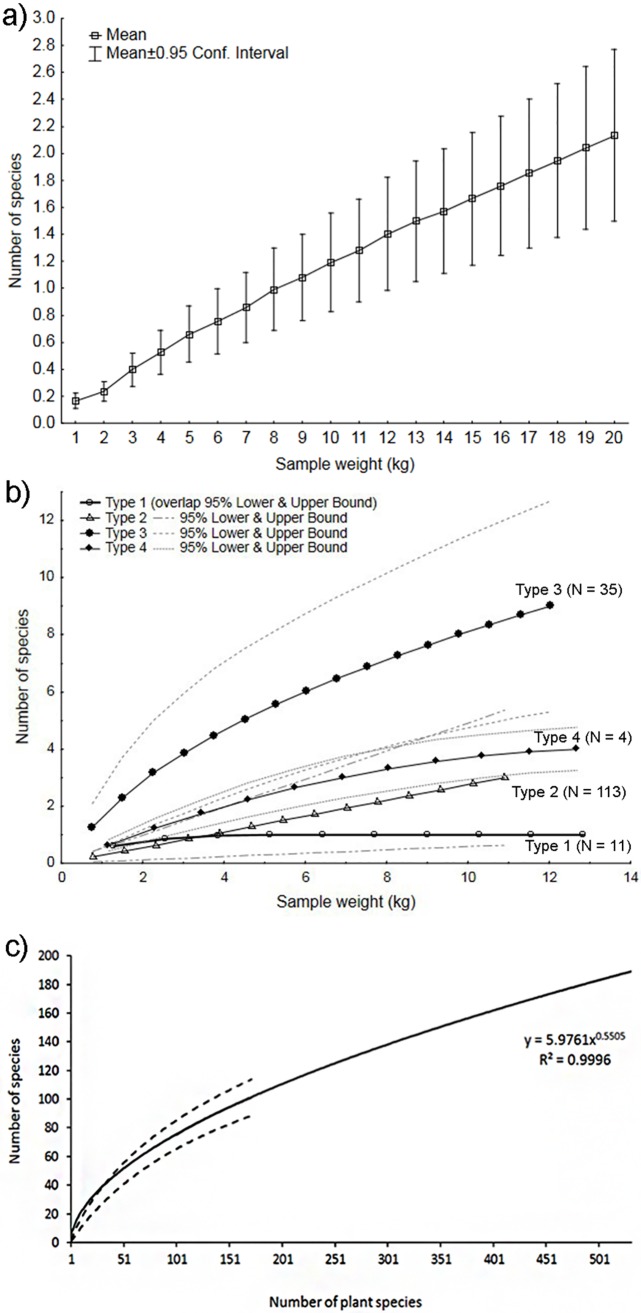
Species accumulation curves for Lepidoptera as functions of sample size. (a) Mean number of lepidopteran species as a function of fruit sample weight for a subset of 33 plant species with sufficiently large samples (1 to 20 kg). (b) Mean number of lepidopteran species per host plant species as a function of fruit sample weight. Confidence intervals (95%) are shown for each of four types of accumulation curve. The number of plant species (N) comprising each curve is also shown. (c) Species richness of Lepidoptera as a function of plant species richness for 163 attacked plant species.

The number of lepidopteran species feeding on a particular plant species increased almost linearly with sample size, from 0.16 ± 0.06 (mean ± 95% CI) in 1-kg samples to 2.13 ± 0.64 in 20-kg samples (Fig 5a). This general trend conceals a diversity of species accumulation curves among individual plant species. We recognized four different patterns of species accumulation including: (1) an asymptote at a single Lepidoptera species per host species, (2) a linear increase in Lepidoptera species per plant species as a function of sample size, (3) an incomplete approach to an asymptote, and (4) an asymptote averaging more than one (specifically, 2–4) Lepidoptera species per host species (Fig 5b).

The estimate of the number of species increased with floristic diversity from 1.64 ± 0.55 (mean ± 95% CI) for a single plant species to 101 ± 12.64 (mean ± 95% CI) for the entire set of 169 plant species analysed, based on the analysis restricted to well sampled plants with ≥5 kg of fruits per species (Fig 5c). A power function extrapolation estimated there should be 189 ± 17 (mean ± 95% CI) Lepidoptera species feeding on the total number of 531 woody plant species sampled in the study (Fig 5c).

**Fig 6:**
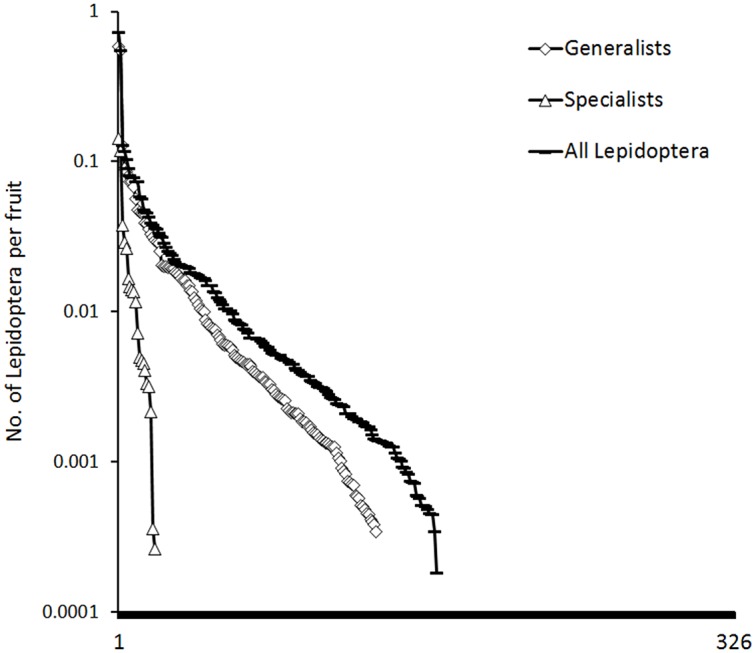
Density of all frugivorous Lepidoptera, and both specialist and generalists, per fruit. Host species are ranked from highest to lowest density for 326 plant species with samples of >1 kg and >50 fruits. Note that all plants to the right of each curve exhibited zero density for the herbivore category in question that cannot be shown on the log scale d y axis.

Most of the 326 plant species exhibited low densities of caterpillars, including both specialists and generalists (Fig 6). One kilogramme of fruits was attacked on average by 1.01 ± 0.18 (mean ± SE) Lepidoptera, and we reared one caterpillar per 89 individual fruits on average, including one generalist per 113 fruits, one specialist per 708 fruits, and one Lepidoptera where specialization could not be determined per 993 fruits.

Only five out of 326 plant species hosted more than one caterpillar per 10 individual fruits (*Pterygota horsfieldii*, *Archidendron aruense*, *Maniltoa schefferi*, *Sterculia schumanniana*, *Xanthophyllum papuanum*; Fig 6), and 55 plant species hosted more than one Lepidoptera per 100 individual fruits. In contrast, 82% of plant species supported densities of less than one Lepidoptera per 100 fruits, suggesting that <1% of fruits were typically attacked.

There was no correlation between lepidopteran density (log (n+1) transformed per 1 kg of fruit or one fruit) and basal area among 218 tree species (Pearson r = -0.007, P = 0.91; Pearson r = -0.02, P = 0.73 respectively). Restricting this analysis to only tree species attacked by Lepidoptera (N = 129) returned the same result (Pearson r = -0.07, P = 0.37; Pearson r = -0.07, P = 0.44 respectively).

## Discussion

### Host specificity

To our knowledge, our study is the first quantitative analysis of host specificity in frugivorous Lepidoptera for tropical forests, showing that their community is strongly dominated by generalist (allofamilial and confamilial) species (77% of all species), whereas specialist species are rare. Comparable data from other studies give inconsistent results. For example, all Lepidoptera species on Dipterocarpaceae in Borneo had confamilial host ranges, i.e. their specificity was low [9]. On the other hand, studies based on massive rearing of frugivorous insects in Kenya [1] found high host specificity in particular families, but this conclusion may be influenced by the effect of sample size. For instance, 57 (58.8%) of the 97 tortricid species reared from fruits in Kenya [4] were recorded from a single plant species, but this number included also 30 species reared as singletons. When using the N = 10 minimum abundance threshold for reared species, as in the present study, the number of monophages decreased to 12 (12.3%) species, similar to the proportion found in the present study where we recorded two (18.2%) monophagous species out of 11 reared tortricids (S1 Table).

## Lineages

There are a few lineages of Lepidoptera that have specialized in feeding on fruit, and these lineages overlap with those that are scavengers, in some cases becoming pests of stored food products [45,46]. Most of the species recorded in this study fall into those lineages. A large study of insects reared from native fruit in Kenya provides an opportunity to evaluate shared lineages. The Kenya program sampled throughout Kenya [1], and thus was not limited to lowland rainforest, as are the PNG samples. Miller and colleagues have published a series of papers on the over 360 Lepidoptera species in the Kenya samples [3–6], with more in preparation.

Blastobasidae: Although Blastobasidae are one of the dominant taxa in both individuals and species in the Kenya samples [3], they are represented by only a few specimens of two species in the PNG samples, one of which is the widespread species *Blastobasis inana* (Butler) [47]. It appears that Blastobasidae are poorly represented in the New Guinea fauna overall, because only several species have been recorded from New Guinea.

Cosmopterigidae: Cosmopteriginae: The closely related genera *Pyroderces* and *Anatrachyntis* (both in need of taxonomic revision), generally considered scavengers [48,49], are represented by different unidentified species in the Kenya and PNG samples. There are also three species of the related genus *Labdia* in the PNG samples.

Crambidae: Cybalomiinae: The genus *Hendecasis* is represented by 2 species in PNG and 4 species in Kenya.

Crambidae: Spilomelinae: Spilomelinae are diverse in both Kenya and New Guinea, and are represented by multiple genera of Spilomelinae in the Kenya and PNG samples. Unfortunately, the lack of a robust phylogeny for the Spilomelinae prevents recognition of possible fruit feeding lineages in common. However, the genus *Prophantis* is represented in Kenya rearings by the well-known coffee pest *Prophantis smaragdina* [50], and in PNG rearings by *Prophantis androstigmata* and an undescribed species.

Lycaenidae: Theclinae: The genus *Deudorix* (sensu lato) is represented by three species in the PNG samples and four species in the Kenya samples. They are known as fruit feeders [51].

Nolidae: Chloephorinae: Several species of the tribe Sarrothripini are in both samples, with the genus *Giaura* represented by one species in PNG and five species in Kenya.

Oecophoridae: Stathmopodinae: The PNG samples include four species of *Stathmopoda* and four species in the Kenya samples.

Pyralidae: Galleriinae: Despite the review by [52], lack of comprehensive classification limits our understanding, but the genus *Lamoria* has been reared in fruit in both Kenya and Papua New Guinea (a species near *Lamoria adaptella* Walker has been reared from seeds of Dipterocarpaceae, [53]), and we have reared three species of the apparently related genus *Tirathaba* in Papua New Guinea (*Tirathaba* is not known to occur in Africa).

Pyralidae: Phycitinae: Phycitinae are diverse in both Kenya and New Guinea, and are represented by multiple genera in the Kenya and PNG samples. Unfortunately, the lack of a robust phylogeny for the Phycitinae ([54]) prevents recognition of possible fruit feeding lineages in common. However, the distinctive genus *Mussidia* stands out because we reared three species from fruit in Kenya (see also [55]), and one species, *Mussidia pectinicornella* Hampson in PNG (see also Genbank KJ668754).

Tineidae: Two widespread genera occur in both the samples [5]; *Erechthias* (Kenya 1 species, PNG 2 species) and *Opogona* (Kenya 1 species, PNG 6 species).

Tortricidae: Olethreutinae: Our PNG results are similar to the Kenya results [4] in that Olethreutinae dominate the Tortricidae in both species and individuals, although Chlidanotinae and Tortricinae are also represented. At least three genera of Olethreutinae occur in both samples; *Cryptaspasma* (Kenya 6 species, PNG 2 species); *Cryptophlebia* (Kenya 3 species, PNG 2 species); and *Lobesia* (Kenya 2 species, PNG 1 species). These genera are known as fruit feeders and some species can be major pests [56].

### Scavengers

Several lineages of Lepidoptera are widely considered to be scavengers (i.e. caterpillars feeding on dead, decaying fruits), and some of these include cosmopolitan pests of stored food products, including some Gelechiidae, Oecophoridae, Pyralidae, and Tineidae [46,57]. It is often difficult to differentiate between larvae feeding on live plant tissue or slightly decaying plant tissue. Some of the genera sampled in PNG that include species recorded as scavengers in the literature include *Labdia* (Cosmopterigidae), *Anatrachyntis* and *Pyroderces* (Cosmopterigidae), *Stathmopoda* (Oecophoridae), *Erechthias* (Tineidae), *Monopis* (Tineidae) and *Opogona* (Tineidae) [19,53]. However, some of these genera include considerable diversity of food habits, so generalizations are difficult.

We identified one specimen of a widespread tropical pest, *Phereoeca uterella* (Walsingham), the plaster bagworm, which is common inside buildings throughout the tropics [58]. Because it may have come from our rearing lab, not from the fruit, we excluded the single specimen from the analysis, but we do consider it a valid record for Papua New Guinea, so have deposited its COI sequence in Genbank (GU695649).

In order to facilitate recognition of the widespread pests of stored products and dried fruit, we have created a DNA barcode library for most of the Lepidoptera in [57]. We have recognized several of these in the Kenya rearings, but none in the Papua New Guinea rearings (Miller, unpublished data in BOLD).

### Overlap between frugivorous and folivorous guilds

We found (Table 1) that even such different resources as fruits (this study) and leaves were jointly exploited by some Lepidoptera species. Although comparable studies analysing overlap between guilds among communities are not available, it has been documented for some Lepidoptera species that they can feed on more than one plant resource. For instance, some species from family Gracillariidae feed on leaves, shoots as well as the fruit surface in avocado [11]. Likewise, species of *Endothenia* spp. (Tortricidae) are borers in the stalks, roots, seeds and fruit of numerous families of flowering plants [59]. *Heleanna physalodes* feed on both flower buds and fruits of different plant species [47] and may be either identical or closely related to the species we found in our study. We can distinguish four groups among the species feeding both on fruits and leaves: (1) leaf chewers rarely using fruits as a minority source of food; (2) species abundant in both guilds, usually also extremely polyphagous; they tend to attack mesocarp causing superficial damage or chewing holes [11], or they may use fruit accidentally [4]; (3) notably, there were no abundant frugivorous species that used leaves only occasionally; (4) species rare in both guilds where we can not specify their food preferences.

Although we are not able to ascertain in most of cases whether our reared lepidopteran species feed on mesocarp or seeds, their impact on fruit fitness may be important in both cases. They may cause fruit loss or reduce seed viability [60] or, as well as other fruit predators, open the way to subsequent attack by pathogens [61] due to mining through the mesocarp or feeding on the surface. These pathogens may make the fruit unpalatable to dispersers through the production of toxins [62]. However, there are also cases where fruit predators were beneficial to their hosts [63] as frugivorous vertebrates preferred fruits infested by insects [64]. There are also predator/pollinator mutualisms of some moths that ensure efficient pollination while feeding on the resulting fruits [65,66].

### Attack rate

The proportion of plant species attacked by Lepidoptera (51.8%) is similar to the 38.4% of attacked plant species found in Kenya [1]. This comparison is based on a similar sampling effort (4,268 fruit samples in PNG vs 3,838 in Kenya), although the number of sampled plant species (531 vs 938) as well as biotopes was higher in Kenya. The attack rate by caterpillars was higher than the 32.5% attacked by weevils (Coleoptera: Curculionidae), obtained from the same data set [26].

### Species diversity and abundance

**Fig 7:**
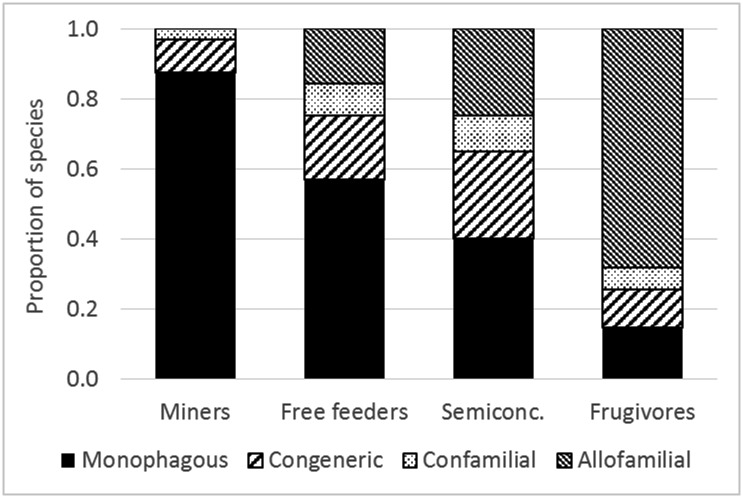
Host specificity of folivorous (mining, freely feeding, and semiconcealed: leaf tying and rolling) and frugivorous guilds of Lepidotera in lowland rainforests of Papua New Guinea.

Although Lepidoptera belong to the most studied herbivore orders in the tropics, this attention is heavily focused on folivorous species [67]. Our studies from the same forests [44,68,69] found on average the following species diversities of Lepidoptera per plant species in different guilds: 1.8 (s.e. 0.25) miners, 15.3 (s.e. 1.26) leaf-tiers and rollers, 9.5 (s.e. 0.90) exposed leaf-chewers, 0.1 (s.e. 0.04) gallers. This suggests that frugivores, with 6.0 species (this study), represent 18% of Lepidoptera diversity, except flower-, root- and wood-feeding species that have not been studied. However, this proportion will decrease with expanding the sampling universe from single plant species to diverse vegetation since frugivores appear to be the least host specific from all Lepidoptera guilds analysed here (Fig 7). The proportion of species feeding on a single host, several congeneric species, confamilial species from different genera, and species from different families is shown for 306 Lepidoptera species reared as ≥10 individuals (folivorous data from [43,44]).

Lepidoptera is the most species rich order among frugivorous species, but their abundance (1.01 individuals per kg; one individual per 89 pieces on fruit) is much lower than in weevils (2.51 individuals per kg; one individual per 33 pieces of fruit; [26]). This low incidence of seed damage as well as very low portion of specialists make it unlikley that frugivorous Lepidoptera play a major role in maintaning of plant diversity due to density-dependent mortality by specailized herbivores. While attack rates might be high enough to influence the pool of seeds in few species, we found also no evidence for density dependence of attacks, which would also argue against Janzen-Connell like processes.

## Supporting information

S1 Table The list of lepidopterans reared from individual plant species. Ns = number of specimen reared, Nb = number of barcodes, Np = number of host plant species used by individuals, S = specificity (categorized as monophagous (M) for species feeding on a single plant species, congeneric (CG), confamilial (CF), and allofamilial (AF) for species feeding on either >1 congeneric species, >1 confamilial genus, or >1 family, respectively).
(PDF)
