# Supplementary material for: Low host specificity and abundance of frugivorous lepidoptera in the lowland rain forests of Papua New Guinea
Source: PLoS One. 2017 Feb 23;12(2):e0171843. doi: 10.1371/journal.pone.0171843 (PMC5322921; doi:10.1371/journal.pone.0171843)
Supplement: S1 Table — Ns = number of specimen reared, Nb = number of barcodes, Np = number of host plant species used by individuals, S = specificity (categorized as monophagous (M) for species feeding on a single plant species, congeneric (CG), confamilial (CF), and allofamilial (AF) for species feeding on either >1 congeneric species, >1 confamilial genus, or >1 family, respectively). (PDF) [file pone.0171843.s001.pdf]

S1 Table. The list of Lepidopterans reared from individual plant species. Ns = number of specimen reared, Nb = number of barcodes, Np = number of host plant species used by individuals , S = specificity (categorized as monophagous (M) for species feeding on a single plant species, congeneric (CG), confamilial (CF), and allofamilial (AF) for species feeding on either >1 congeneric species, >1 confamilial genus, or >1 family, respectively

Lepidoptera ID	Ns	Nb	Np	S	Plant family	Plant species
Blastobasidae						
Blastobasinae						
Blastobasis inana	3	3	3		Annonaceae	*Goniothalamus aruensis*
					Loganiaceae	*Strychnos minor*
					Rubiaceae	*Mastixiodendron pachyclados*
Neoblastobasis sp1	10	3	3	AF	Anacardiaceae	*Semecarpus cassuvium*
					Euphorbiaceae	*Macaranga aleuritoides*
					Rubiaceae	*Versteegia cauliflora*
Brachodidae						
Brachodinae						
Miscera basichrysa extensa	5	5	2		Arecaceae	*Calamus longipinna*
					Meliaceae	*Dysoxylum arborescens*
Carposinidae						
Carposina sp1	2	2	1		Rubiaceae	*Versteegia cauliflora*
Cosmopterigidae						
Chrysopeleiinae						
Gn sp1	4	1	1		Araceae	*Pothos rumphii*
Cosmopteriginae						
Anatrachyntis nr (not) badia	27	6	5	AF	Arecaceae	*Hydriastele microspadix*
					Asparagaceae	*Cordyline terminalis*
					Asparagaceae	*Dracaena angustifolia*
					Lamiaceae	*Premna obtusifolia*
					Polygalaceae	*Xanthophyllum papuanum*
Labdia albimaculella	1	1	1		Dipterocarpaceae	*Pterocarpus indicus*
Labdia sp1	5	4	5		Arecaceae	*Licuala beccariana*
					Fabaceae	*Inocarpus fagifer*
					Meliaceae	*Dysoxylum confertiflorum*
					Myristicaceae	*Myristica buchneriana*
					Putranjivaceae	*Drypetes longifolia*
Labdia sp2	14	1	10	AF	Euphorbiaceae	*Cleistanthus myrianthus**
Labdia sp3	1	1	1		Euphorbiaceae	*Cleistanthus myrianthus*
Labdia sp4	17	3	3	AF	Icacinaceae	*Medusanthera laxiflora*
					Myristicaceae	*Myristica buchneriana*

**Lepidoptera ID**	**Ns**	**Nb**	**Np**	**S**	**Plant family**	**Plant species**
					Solanaceae	*Solanum oliverianum*
Pyroderces sp1	95	10	7	AF	Araceae	*Pothos rumphii**
Crambidae						
Spilomelinae						
Cirrhochrista nr brizoalis	1	1	1		Moraceae	*Ficus botryocarpa*
Cirrhochrista sp1	1	1	1		Moraceae	*Ficus austrina*
Conogethes pluto	10	6	2	AF	Fabaceae	*Maniltoa megalocephala*
					Zingiberaceae	*Pleuranthodium racemigerum*
Conogethes semifascialis	17	4	7	AF	Malvaceae	*Pterygota horsfieldii**
cf Herpetogramma	2	2	1		Zingiberaceae	*Pleuranthodium racemigerum*
Lipararchis sp1	3	2	1		Araceae	*Epipremnum pinnatum*
Nacoleia octasema	3	3	1		Heliconiaceae	*Heliconia papuana*
Pagyda botydalis	9	1	2		Lamiaceae	*Callicarpa farinosa*
					Lamiaceae	*Callicarpa pentandra*
Pagyda salvalis	4	2	2		Lamiaceae	*Vitex cofassus*
					Marantaceae	*Donax canniformis*
Piletocera nr aegimiusalis	1	1	1		Sapindaceae	*Lepidopetalum micans*
Piletocera violascens	3	2	3		Zingiberaceae	*Pleuranthodium racemigerum*
					Fabaceae	*Millettia pinnata*
					Lecythidaceae	*Planchonia papuana*
Prophantis androstigmata	4	3	3		Elaeocarpaceae	*Elaeocarpus amplifolius*
					Loganiaceae	*Neuburgia corynocarpa*
					Rubiaceae	*Randia decora*
Prophantis sp1	13	7	1	M	Rubiaceae	*Pavetta platyclada*
Erebidae						
Tolpiodes sp1	1	1	1		Phyllanthaceae	*Aporosa papuana*
Arctiinae						
Caeneressa bernhardii	1	1	1		Gnetaceae	*Gnetum gnemon*
Chamaita sp1	1	0	1		Lamiaceae	*Faradaya splendida*
Gelechiidae					Rubiaceae	*Mussaenda cylindrocarpa*
Gn sp1	13	3	1	M	Sapindaceae	*Tristiropsis acutangula*
Gn sp2	217	6	11	AF	Fabaceae	*Archidendron aruense**
Dichomeridinae						

Lepidoptera ID	Ns	Nb	Np	S	Plant family	Plant species
Dichomeris thryptica	1	1	1		Sapotaceae	*Chrysophyllum roxburghii*
Gelechiinae						
Ardozyga sp1	1	1	1		Euphorbiaceae	*Antidesma contractum*
Gracillariidae						
Gn sp1	15	5	3	CF	Fabaceae	*Archidendron aruense*
					Fabaceae	*Archidendron glabrum*
					Fabaceae	*Inocarpus fagifer*
Gn sp2	17	1	8	AF	Fabaceae	*Maniltoa plurijuga**
Gracillariinae						
Conopomorpha cramerella	16	4	3	AF	Euphorbiaceae	*Ryparosa javanica*
					Fabaceae	*Maniltoa psilogyne*
					Sapindaceae	*Pometia pinnata*
Conomorpha sp1	1	1	1		Euphorbiaceae	*Ryparosa javanica*
Conomorpha sp2	1	1	1		Fabaceae	*Maniltoa psilogyne*
Heliodinidae?						
Gn sp1	34	6	1	M	Myrtaceae	*Syzygium gonatanthum*
Immidae						
Moca congrualis	1	1	1		Burseraceae	*Canarium vitiense*
Lecithoceridae						
Lecithocera sp1	7	3	5		Annonaceae	*Pseuduvaria mollis*
					Annonaceae	*Uvaria rosenbergiana*
					Lamiaceae	*Callicarpa longifolia*
					Lamiaceae	*Gmelina moluccana*
					Sapindaceae	*Harpullia crustacea*
Gn sp1	5	3	2		Fabaceae	*Inocarpus fagifer*
					Myristicaceae	*Horsfieldia basifissa*
Gn sp2	20	9	3	AF	Araliaceae	*Osmoxylon novo-guineense*
					Meliaceae	*Chisocheton gliroides*
					Monimiaceae	*Steganthera hirsuta*
Gn sp3	17	4	4	AF	Lamiaceae	*Clerodendrum tracyanum*
					Myristicaceae	*Myristica fatua*
					Moraceae	*Prainea papuana*
					Rosaceae	*Prunus schlechteri*

Lepidoptera ID	Ns	Nb	Np	S	Plant family	Plant species
Gn sp4	1	1	1		Euphorbiaceae	*Omphalea papuana*
Gn sp5	12	2	5	AF	Araceae	*Epipremnum pinnatum*
					Euphorbiaceae	*Macaranga aleuritoides*
					Euphorbiaceae	*Macaranga bifoveata*
					Meliaceae	*Dysoxylum confertiflorum*
					Moraceae	*Ficus pachyrrhachis*
Gn sp6	2	2	1		Menispermaceae	*Pycnarrhena novoguineensis*
Gn sp7	1	1	1		Euphorbiaceae	*Macaranga bifoveata*
Lycaenidae						
Polymmatinae						
Jamides cyta	10	5	2	CG	Myrtaceae	*Syzygium amplum*
					Myrtaceae	*Syzygium longipes*
Theclinae						
Deudorix diovis	11	5	4	CF	Sapindaceae	*Alectryon ferrugineum*
					Sapindaceae	*Cupaniopsis stenopetala*
					Sapindaceae	*Harpullia crustacea*
					Sapindaceae	*Lepidopetalum micans*
Deudorix epirus	13	4	5	AF	Fabaceae	*Maniltoa lenticellata*
					Fabaceae	*Maniltoa psilogyne*
					Pandaceae	*Galearia celebica*
					Sapindaceae	*Harpullia longipetala*
					Thymelaeaceae	*Phaleria macrocarpa*
Deudorix littoralis	4	4	2		Dipterocarpaceae	*Vatica papuana*
					Fabaceae	*Archidendron glabrum*
Noctuidae						
Amphipyrinae						
Spodoptera litura	1	1	1		Apocynaceae	*Tabernaemontana sp.*
Spodoptera pecten	1	1	1		Arecaceae	*Caryota rumphiana*
Heliothinae						
Helicoverpa assulta	2	1	1		Solanaceae	*Solanum americanum*
Nolidae						
Chloephorinae						
Etanna vittalis	2	2	1		Anacardiaceae	*Spondias dulcis*

Lepidoptera ID	Ns	Nb	Np	S	Plant family	Plant species
Nolinae						
Nola sp1	1	0	1		Myristicaceae	*Horsfieldia basifissa*
Sarrothripinae						
Giaura nr leucophaea	13	5	1	M	Malvaceae	*Pterygota horsfieldii*
Gyrtothripa quadrifera	27	9	4	AF	Fabaceae	*Gigasiphon schlechteri*
					Malvaceae	*Sterculia shillinglawii*
					Malvaceae	*Sterculia schumanniana*
					Thymelaeaceae	*Phaleria macrocarpa*
Gyrtothripa verecunda	11	6	1	M	Malvaceae	*Sterculia schumanniana*
Oecophoridae						
Stathmopodinae						
Stathmopoda nr periclina	4	3	2		Lecythidaceae	*Barringtonia calyptrocalyx*
					Myrtaceae	*Syzygium malaccense*
Stathmopoda nr tetrazyga	3	2	2		Rubiaceae	*Versteegia cauliflora*
					Dipterocarpaceae	*Vatica papuana*
Stathmopoda sp1	1	1	1		Lauraceae	*Litsea collina*
Stathmopoda sp2	3	3	2		Lauraceae	*Litsea globosa*
					Lauraceae	*Litsea timoriana*
Stathmopoda sp3	8	5	1		Arecaceae	*Hydriastele microspadix*
Stathmopoda sp4	2	2	1		Lauraceae	*Litsea collina*
Stathmopoda sp5	4	3	1		Lauraceae	*Litsea collina*
Pterophoridae						
Pterophorinae						
Xyroptila nr peltastes	1	1	1		Rubiaceae	*Versteegia cauliflora*
Pyralidae						
Gn sp1	1	1	1		Sapindaceae	*Pometia pinnata*
Cybalomiinae						
Hendecasis melalophalis	5	3	1		unknown	
Hendecasis nr duplifascialis	6	4	1		unknown	
Galleriinae						
Lamoria adaptella complex	13	9	4	AF	Fabaceae	*Intsia bijuga*
					Marantaceae	*Phrynium macrocephalum*
					Marantaceae	*Phrynium pedunculatum*

Lepidoptera ID	Ns	Nb	Np	S	Plant family	Plant species
					Rubiaceae	*Versteegia cauliflora*
Microchlora sp.n. nr bileneella	1	1	1		Myristicaceae	*Horsfieldia sylvestris*
Picrogama semifoedalis	1	1	1		Malvaceae	*Pterygota horsfieldii*
Tirathaba pseudocomplana	29	6	5	AF	Arecaceae	*Caryota rumphiana*
					Fabaceae	*Inocarpus fagifer*
					Moraceae	*Ficus congesta*
					Moraceae	*Ficus hispidioides*
					Moraceae	*Ficus pachyrrhachis*
Tirathaba nr rufivena	1	1	1		Meliaceae	*Aphanamixis polystachya*
Tirathaba ruptilinea	14	8	5	AF	Araliaceae	*Osmoxylon novo-guineense*
					Euphorbiaceae	*Cleistanthus myrianthus*
					Moraceae	*Ficus congesta*
					Moraceae	*Ficus pachyrrhachis*
					Sapindaceae	*Harpullia longipetala*
Phycitinae						
Assara seminivale	19	13	5	AF	Dipterocarpaceae	*Vatica papuana*
					Fabaceae	*Inocarpus fagifer*
					Rubiaceae	*Canthium cymigerum*
					Rubiaceae	*Canthium longiflorum*
					Lamiaceae	*Teijsmanniodendron bogoriense*
Assara nr subarcuella	11	6	1	M	Dipterocarpaceae	*Vatica papuana*
Faveria nr dasyptera	3	1	2		Lauraceae	*Cryptocarya caudata*
					Verbenaceae	*Premna obtusifolia*
Mussidia cf pectinicornella	13	8	3	CF	Fabaceae	*Intsia bijuga*
					Fabaceae	*Kingiodendron novoguineense*
					Fabaceae	*Millettia pinnata*
Pyralinae						
Gn sp1	4	3	1		Lythraceae	*Lagerstroemia piriformis*
Schreckensteiniidae						
Gn sp1	37	2	2	AF	Maesaceae	*Maesa haplobotrys*
					Marantaceae	*Phrynium macrocephalum*
Thyrididae						
Siculonidae						

Lepidoptera ID	Ns	Nb	Np	S	Plant family	Plant species
Picrostomastis inductalis	3	3	1		Euphorbiaceae	*Glochidion harveyanum*
Tineidae						
Erechtiinae						
Erechthias sp1	108	14	44	AF	Lamiaceae	*Premna obtusifolia**
Erechthias sp2	2	1	1		Polygalaceae	*Xanthophyllum papuanum*
Erechthias sp3	7	5	1		Sapindaceae	*Ganophyllum falcatum*
Pyloetis sp1	1	1	1		Dipterocarpaceae	*Pterocarpus indicus*
Hapsiferinae						
Tiquadra sp1	10	7	7	AF	Fabaceae	*Inocarpus fagifer**
Trachycentra chlorogramma	7	4	4		Dipterocarpaceae	*Vatica papuana*
					Lecythidaceae	*Barringtonia calyptrocalyx*
					Marantaceae	*Phrynium macrocephalum*
					Rubiaceae	*Versteegia cauliflora*
Hieroxestinae						
Opogona taochroa	7	8	6		Arecaceae	*Arenga microcarpa**
Opogona sp1	1	1	1		Meliaceae	*Dysoxylum arborescens*
Opogona sp2	2	1	1		Fabaceae	*Archidendron glabrum*
					Heliconiaceae	*Heliconia papuana*
Opogona sp3	3	3	3		Malvaceae	*Microcos argentata*
					Icacinaceae	*Polyporandra scandens*
					Menispermaceae	*Pycnarrhena novoguineensis*
Opogona sp4	27	3	6	AF	Heliconiaceae	*Heliconia papuana**
Opogona sp5	8	3	3		Fabaceae	*Maniltoa psilogyne*
					Meliaceae	*Dysoxylum arborescens*
					Myrtaceae	*Syzygium trivene*
Opogona sp6	16	1	8	AF	Lecythidaceae	*Planchonia papuana**
Meessiinae						
Gn sp1	2	2	2		Araceae	*Amorphophallus campanulatus*
					Icacinaceae	*Gomphandra papuana*
Tineinae						
Monopis pentadisca	151	5	33	AF	Gnetaceae	*Gnetum gnemonoides**

Lepidoptera ID	Ns	Nb	Np	S	Plant family	Plant species
Phereoeca uterella	1	1	1		Proteaceae	*Helicia latifolia*
Tortricidae						
Gn sp1	2	1	1		Lauraceae	*Cryptocarya massoy*
Gn sp2	2	0	2		Sapindaceae	*Cupaniopsis macropetala*
					Icacinaceae	*Polyporandra scandens*
Gn sp3	3	0	1		Icacinaceae	*Gonocaryum litorale*
Chlidanotinae						
Caenognosis incisa	1	1	1		Sapotaceae	*Chrysophyllum roxburghii*
Polylopha sp1	3	2	1		Lauraceae	*Litsea timoriana*
Hilarographa nr muluana	8	8	2		Marantaceae	*Phrynium macrocephalum*
					Marantaceae	*Phrynium pedunculatum*
Hilarographa sp1	175	5	7	AF	Loganiaceae	*Strychnos minor**
Hilarographa sp2	12	5	1	M	Rubiaceae	*Versteegia cauliflora*
Olethreutinae						
Andrioplecta nr leucodora	72	7	13	AF	Fabaceae	*Kingiodendron alternifolium**
Cryptaspasma brachyptycha	8	3	3		Lauraceae	*Cryptocarya depressa*
					Myrtaceae	*Syzygium amplum*
					Myrtaceae	*Syzygium trivene*
Cryptaspasma sp1	1	1	1		Lauraceae	*Litsea collina*
Cryptophlebia sp1	21	10	1	M	Sapindaceae	*Tristiropsis acutangula*
Cryptophlebia sp2	1	1	1		Rubiaceae	*Versteegia cauliflora*
Cydia sp1	30	7	3	CF	Fabaceae	*Maniltoa psilogyne*
					Fabaceae	*Millettia pinnata*
					Fabaceae	*Millettia pinnata*
Cydia sp2	2	0	1		Clusiaceae	*Garcinia assugu*
Demeijerella sp.n.	19	6	4	AF	Arecaceae	*Hydriastele microspadix*
					Marantaceae	*Donax canniformis*
					Rubiaceae	*Psychotria leptothyrsa*
					Sapindaceae	*Tristiropsis acutangula*
Gnathmocerodes labidophora	7	5	5		Fabaceae	*Archidendron glabrum*
					Lecythidaceae	*Barringtonia apiculata*
					Lecythidaceae	*Barringtonia calyptrocalyx*
					Lecythidaceae	*Planchonia papuana*

Lepidoptera ID	Ns	Nb	Np	S	Plant family	Plant species
					Vitaceae	*Cayratia schumanniana*
Heleanna nr physalodes	9	6	1		Euphorbiaceae	*Pimelodendron amboinicum*
Helictophanes nr scambodes	24	6	3	CG	Meliaceae	*Dysoxylum arborescens*
					Meliaceae	*Dysoxylum excelsum*
					Meliaceae	*Dysoxylum pettigrewianum*
Helictophanes nr prospera	3	2	3		Malvaceae	*Microcos argentata*
					Meliaceae	*Dysoxylum arborescens*
					Meliaceae	*Dysoxylum pettigrewianum*
Helictophanes nr uberana	5	5	3		Meliaceae	*Aglaia argentea*
					Meliaceae	*Aphanamixis polystachya*
					Meliaceae	*Chisocheton sayeri*
Helictophanes sp1	2	1	2		Meliaceae	*Aglaia sp.*
					Meliaceae	*Sandoricum koetjape*
Helictophanes sp2	2	1	1		Lamiaceae	*Vitex cofassus*
Lobesia sp1	2	1	2		Euphorbiaceae	*Melanolepis multiglandulosa*
					Lamiaceae	*Clerodendrum tracyanum*
Opiorhabda deceptor	2	2	1		Myrtaceae	*Syzygium coalitum*
Parammene sp1	1	1	1		Sapindaceae	*Mischocarpus sundaicus*
Thaumatotibia nr zophophanes	155	16	22	AF	Malvaceae	*Pterygota horsfieldii**
Gn sp1	4	1	1		Arecaceae	*Hydriastele microspadix*
Gn sp2	4	0	4		Burseraceae	*Canarium vitiense*
					Fabaceae	*Inocarpus fagifer*
					Myristicaceae	*Horsfieldia irya*
					Polygalaceae	*Xanthophyllum papuanum*
Gn sp3	4	2	1		Clusiaceae	*Garcinia assugu*
Tortricinae						
Adoxophyes sp.n. nr orana	1	1	1		Euphorbiaceae	*Cleistanthus myrianthus*
Adoxophyes thoracica	1	1	1		Elaeocarpaceae	*Elaeocarpus amplifolius*
Adoxophyes tripselia	1	1	1		Araliaceae	*Polyscias verticillata*
Adoxophyes sp1	1	1	1		Oleaceae	*Chionanthus sessiliflorus*
Epitrichosma nr metreta	11	3	8	AF	Asparagaceae	*Cordyline terminalis**
Isodemis nr stenotera	1	1	1		Lamiaceae	*Clerodendrum tracyanum*
Gn sp1	80	8	11	AF	Icacinaceae	*Gonocaryum litorale**

Lepidoptera ID		Ns	Nb	Np	S	Plant family	Plant species
	Gn sp2	19	5	4	AF	Proteaceae	*Helicia latifolia*
						Sapindaceae	*Harpullia crustacea*
						Sapindaceae	*Tristiropsis acutangula*
						Combretaceae	*Terminalia complanata*
Xyloricitdae							
	Gn sp1	15	5	3	CG	Meliaceae	*Dysoxylum arborescens*
						Meliaceae	*Dysoxylum confertiflorum*
						Meliaceae	*Dysoxylum pettigrewianum*
Lepidoptera							
	Gn sp1	3	3	2		Euphorbiaceae	*Cleistanthus myrianthus*
						Lamiaceae	*Clerodendrum tracyanum*
	Gn sp2	5	2	2		Malvaceae	*Microcos argentata*
						Malvaceae	*Microcos grandiflora*
	Gn sp3	21	4	2	AF	Sapindaceae	*Cupaniopsis stenopetala*
						Burseraceae	*Canarium vitiense*
	Gn sp4	4	3	1		Arecaceae	*Hydriastele microspadix*
	Gn sp5	2	2	1		Meliaceae	*Dysoxylum brevipaniculum*
	Gn sp6	2	2	1		Arecaceae	*Arenga microcarpa*
	Gn sp7	5	2	4		Burseraceae	*Canarium vitiense*
						Euphorbiaceae	*Pimelodendron amboinicum*
						Lamiaceae	*Vitex cofassus*
						Myristicaceae	*Horsfieldia sylvestris*
	Gn sp8	7	4	2		Meliaceae	*Chisocheton sayeri*
						Meliaceae	*Chisocheton sp.*
	Gn sp9	2	1	1		Hypoxidaceae	*Curculigo recurvata*
	Gn sp10	2	2	1		Zingiberaceae	*Alpinia peekelii*
	Gn sp11	4	1	1		Myrtaceae	*Syzygium trachyanthum*
Microlepidoptera							
	Gn sp1	1	1	1		Myristicaceae	*Endocomia macrocoma*
	Gn sp2	1	1	1		Euphorbiaceae	*Macaranga aleuritoides*

Notes: Ns = number of specimen reared, Nb = number of barcodes, Np = number of host plant species used by individuals , S = specificity (categorized as monophagous (M) for species feeding on a single plant species, congeneric (CG), confamilial (CF), and allofamilial (AF) for species feeding on either >1 congeneric species, >1 confamilial genus, or >1 family, respectively)
